# Oncogenic Features of PHF8 Histone Demethylase in Esophageal Squamous Cell Carcinoma

**DOI:** 10.1371/journal.pone.0077353

**Published:** 2013-10-11

**Authors:** Xiujing Sun, Jihui Julia Qiu, Shengtao Zhu, Bangwei Cao, Lin Sun, Sen Li, Peng Li, Shutian Zhang, Shuo Dong

**Affiliations:** 1 Department of Medicine and Dan L. Duncan Cancer Center, Baylor College of Medicine, Houston, Texas, United States of America; 2 Department of Gastroenterology, Beijing Friendship Hospital Affiliated to Capital Medical University, Beijing, China; 3 Department of Pathology and Laboratory Medicine, Temple University School of Medicine, Philadelphia, Pennsylvania, United States of America; 4 Department of Oncology, Beijing Friendship Hospital Affiliated to Capital Medical University, Beijing, China; National Cancer Institute, National Institutes of Health, United States of America

## Abstract

Esophageal cancer is the sixth leading cause of cancer-related deaths worldwide. It has been reported that histone demethylases are involved in the carcinogenesis of certain types of tumors. Here, we studied the role of one of the histone lysine demethylases, plant homeodomain finger protein 8 (PHF8), in the carcinogenesis of esophageal squamous cell carcinoma (ESCC). Using short hairpin RNA via lentiviral infection, we established stable ESCC cell lines with constitutive downregulation of PHF8 expression. Knockdown of PHF8 in ESCC cells resulted in inhibition of cell proliferation and an increase of apoptosis. Moreover, there were reductions of both anchorage-dependent and -independent colony formation. *In vitro* migration and invasion assays showed that knockdown of PHF8 led to a reduction in the number of migratory and invasive cells. Furthermore, downregulation of PHF8 attenuated the tumorigenicity of ESCC cells *in vivo*. Taken together, our study revealed the oncogenic features of PHF8 in ESCC, suggesting that PHF8 may be a potential diagnostic marker and therapeutic target for ESCC.

## Introduction

Esophageal cancer is the sixth leading cause of cancer-related deaths worldwide, claiming approximately 406,000 lives annually. There are approximately 481,000 new cases of esophageal cancer diagnosed each year [1]. The highest esophageal cancer-related morbidity and mortality rates have been registered in China.

Based on histology, more than 90% of esophageal cancers can be categorized into either squamous cell carcinoma or adenocarcinoma. Esophageal squamous cell carcinoma (ESCC) accounts for most cases of esophageal cancer worldwide [2]. The prognosis of patients diagnosed with ESCC remains unsatisfactory despite recent improvements in evidence-based management and novel treatments. A lack of comprehension of the underlying mechanism(s) including carcinogenesis as well as a paucity of sensitive/specific molecular markers for early diagnosis are factors hindering appropriate prognostic classification of ESCC patients.

Previous studies including our own have shown that aberrant epigenetic alteration by DNA cytosine methylation plays a crucial role in the development of ESCC [3], [4], [5]. Overexpression/mutation of histone lysine demethylases (KDMs) has been implicated in tumor initiation and progression [6], [7]. These enzymes may be new therapeutic targets in oncology, and compounds inhibiting their activities are of considerable interest as novel anticancer agents [8]. Thus far, only one of the KDMs, KDM4C/GASC1, has been found to be modified in esophageal cancer cell lines and might be involved in cancer development [9]. To further analyze the roles of KDM genes, we investigated the role of a plant homeodomain finger protein 8 (PHF8) in the pathogenesis and progression of ESCC.

PHF8 is a member of one of the most recently discovered families of KDMs, and demethylates H3K9me1/2, H3K27me2, and H4K20me1 [10], [11], [12]. It has been shown that PHF8 is associated with X-linked mental retardation, a condition characterized by mild mental retardation, a cleft lip and palate, and facial dysmorphism [13]. Recent studies have described multiple roles for PHF8 in activation of gene transcription and cell-cycle control, and knockdown of PHF8 leads to a decrease of cell proliferation [10], [11], [14], [15]. Furthermore, PHF8 is overexpressed in prostate cancer with effects on cell proliferation, migration, and invasion [16]. In this study, we investigated the role of PHF8 in ESCC and found that PHF8 has many oncogenic features including promotion of ESCC cell proliferation and tumor growth as well as ESCC cell migration and invasion abilities. Importantly, our study indicates that expression of PHF8 is required for maintenance of ESCC cell proliferation.

## Results

### PHF8 Promotes Proliferation of ESCC Cells

**Figure 1:**
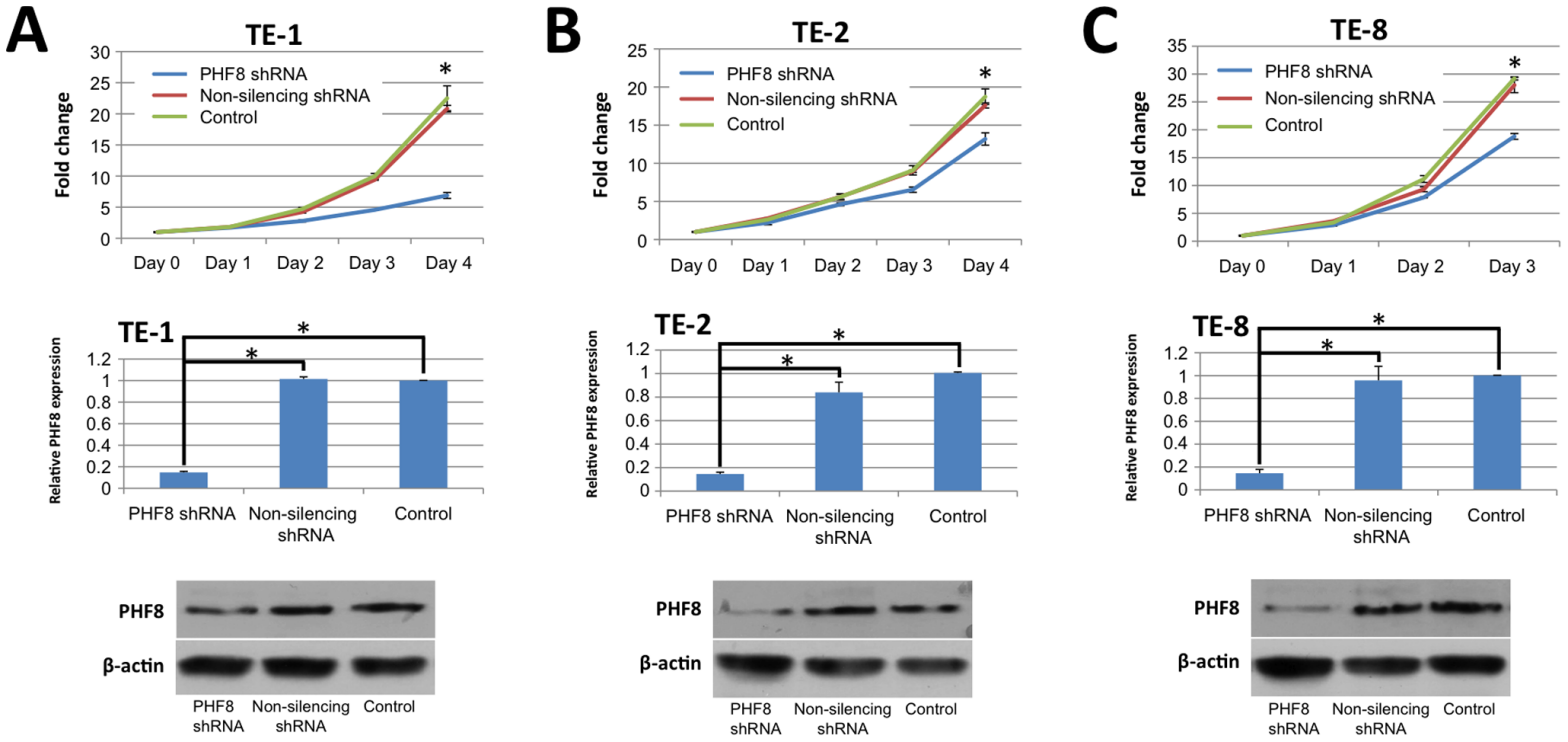
PHF8 promotes ESCC cell proliferation. ESCC cell lines TE-1 (**A**), TE-2 (**B**), and TE-8 (**C**) stably expressing PHF8 shRNA or non-silencing shRNA, or control cells were seeded in triplicate in a 96-well plate. MTS was added to the cultures and analyzed by a microplate photometer at the indicated times. Data are the means ± SD of three independent experiments (*P<0.05). Downregulation of PHF8 mRNA and protein expression induced by PHF8 shRNA was confirmed by real-time quantitative PCR and western blotting, respectively. GAPDH and β-actin served as endogenous controls.

It has been reported that PHF8 regulates HeLa cancer cell growth via its KDM activity [10], [11]. We therefore hypothesized that PHF8 may have important effects on tumorigenesis in esophageal squamous cell carcinoma (ESCC). To test our hypothesis, we used a lentivirus-mediated short hairpin RNA (shRNA) strategy to downregulate PHF8 expression in a series of ESCC cell lines (TE-1, TE-2, and TE-8). Using real-time quantitative PCR and western blot analysis, we confirmed substantial reductions of PHF8 expression levels in these cell lines by shRNA-mediated knockdown (Figure 1). Next, we performed MTS assays to measure cell proliferation [17]. Following knockdown of PHF8 expression, we found significant inhibition of the proliferation of ESCC cells (TE-1, TE-2, and TE-8), indicating that PHF8 positively regulated the proliferation of ESCC cells (Figure 1). To exclude potential off-target effects, the same experiments were performed using another PHF8 shRNA (PHF8 shRNA-2), which showed similar inhibitive effects (Figure S1). Among the ESCC cell lines, TE-1 is well differentiated, whereas TE-2 and TE-8 are poorly and moderately differentiated, respectively. The inhibitive effect on TE-1 cell proliferation was more evident than that observed in TE-2 and TE-8 cells. The inhibition rates of TE-1, TE-2, and TE-8 cells were 66.8%, 25.2%, and 32.8%, respectively (Figure 1A–C).

### Both Anchorage-dependent and -independent Growth of ESCC Cells are Suppressed Following Knockdown of PHF8

Next, we performed colony formation assays, including a plate colony formation assay (for anchorage-dependent growth) and soft agar colony formation assay (for anchorage-independent growth), which reflect the proliferation and self-renewal characteristics of cells.

**Figure 2:**
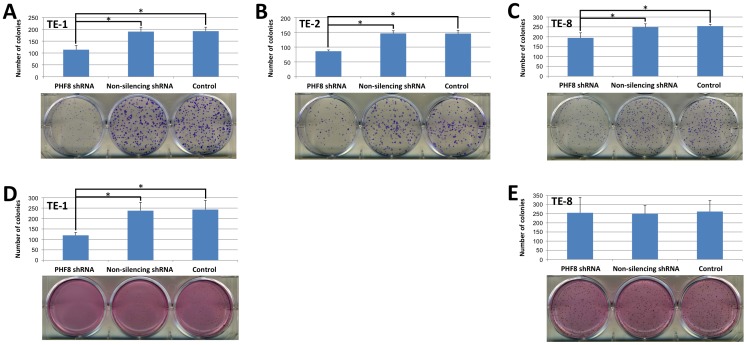
PHF8 facilitates ESCC cell colony formation. ESCC cell lines TE-1, TE-2, and TE-8 stably expressing PHF8 or non-silencing shRNA, or control cells were seeded in triplicate in a six-well plate for plate colony formation and soft agar colony formation assays. The cells were stained with Giemsa or INT at the end of the experiments to count the colonies. Data are the means ± SD of three independent experiments (*P<0.05). Representative images are shown. (**A**–**C**) Results of the plate colony formation assay of TE-1, TE-2, and TE-8 cells, respectively. (**D**, **E**) Results of the soft agar colony formation assay of TE-1 and TE-8 cells, respectively, in which, we noted that the smaller colonies were observed in TE-1 cells, in comparison with TE-8 cells.

We first performed the plate colony formation assay to determine the effect of PHF8 on anchorage-dependent growth of ESCC cells. The tested ESCC cell lines formed colonies on the surface of culture dishes at various efficiencies. The average numbers of colonies formed by the three cell lines are presented in Table S1A. TE-8 cells had the highest colony formation efficiency (50.8%), whereas TE-1 and TE-2 cells had moderate (38.4%) and the lowest (29.1%) colony formation efficiencies, respectively. All of the three cell lines with PHF8 knockdown formed significantly fewer and smaller colonies than those formed by cells expressing non-silencing shRNA or control cells (Figure 2A–C). The inhibition rates of TE-1, TE-2, and TE-8 cells were 40.6%, 40.8%, and 23.4%, respectively. These results suggest that PHF8 is involved in anchorage-dependent growth of ESCC cells and knockdown of PHF8 reduces the anchorage-dependent colony formation ability of ESCC cells.

Many tumor cell types are adherent and only grow while attached to a solid support. Thus, most tumor cells can form colonies in culture dishes, which reflect their anchorage-dependent growth ability. However, tumor cells can also acquire the ability to grow as colonies in semi-solid media, which reflects their ability for anchorage-independent growth. We thus performed a modified soft agar colony formation assay to explore the effect of PHF8 on anchorage-independent growth of ESCC cells. Soft agar is a semi-solid support medium that reduces cell movement and allows individual cells to develop into colonies. As shown in Figure 2D and 2E, ESCC cell lines TE-1 and TE-8, but not TE-2 (data not shown), were able to form colonies in the soft agar assay. The average numbers of colonies are shown in Table S1B. The colony formation efficiencies of TE-1 and TE-8 cells were 12.1% and 13.1%, respectively. Consistent with the results of the plate colony formation assay, PHF8 knockdown in TE-1 cells resulted in significantly fewer and smaller colonies than those formed by cells expressing non-silencing shRNA or control cells. The inhibition rate of colony formation was 50.8% (Figure 2D). However, there was no significant difference in the colony formation of TE-8 cells (Figure 2E).

### PHF8 Suppresses Apoptosis of ESCC Cells *in vitro*

**Figure 3:**
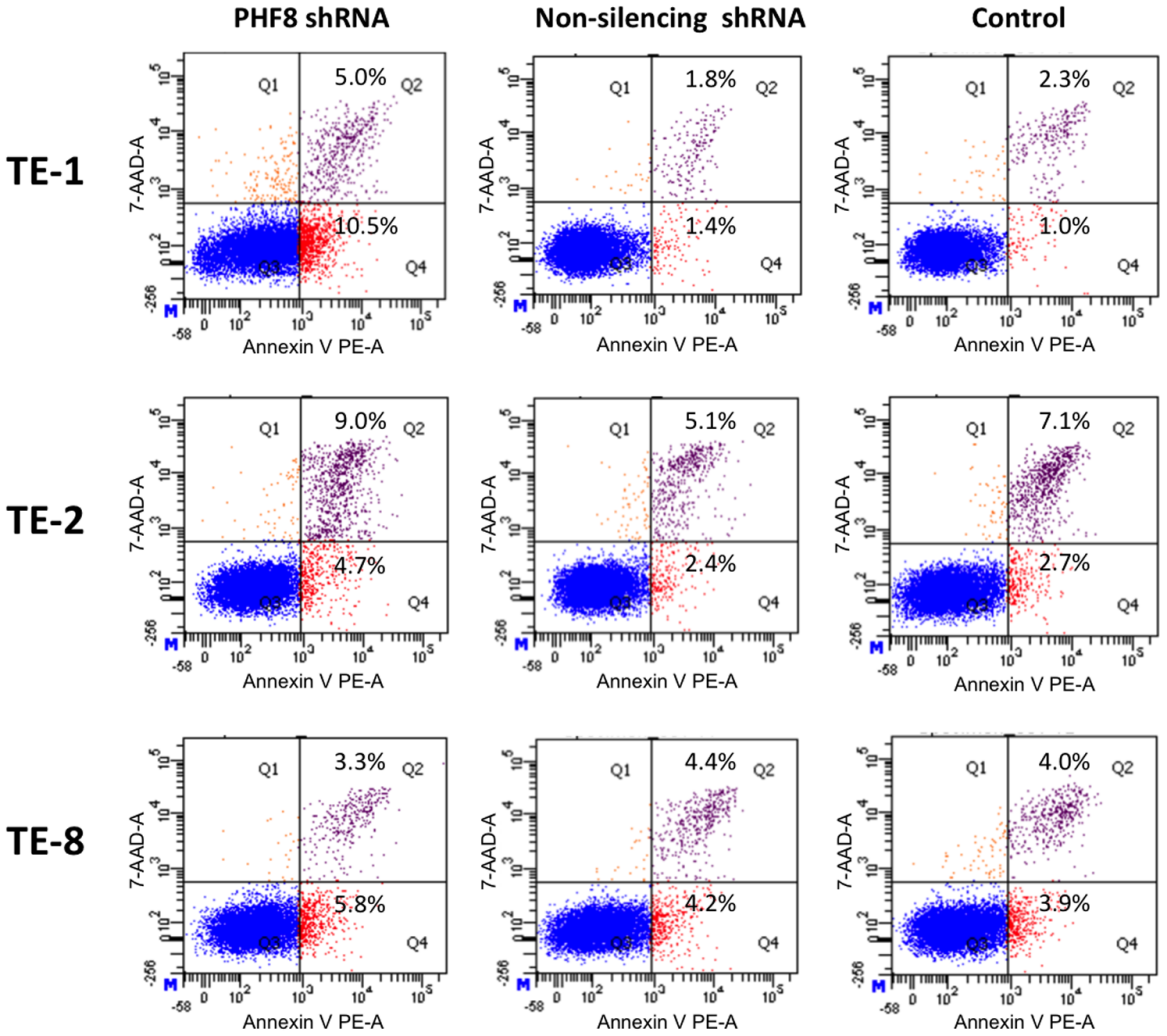
PHF8 inhibits ESCC cell apoptosis. ESCC cells stably expressing PHF8 or non-silencing shRNA, or control cells were stained with annexin V-PE and 7-AAD, and then analyzed by an LSRFortessa. Annexin V-PE is a sensitive probe for identification of apoptotic cells, whereas 7-AAD is a standard flow cytometric viability probe used to detect non-viable cells. Cells that stain positive for both annexin V-PE and 7-AAD are either in late apoptosis or are non-viable (Q2). Early apoptotic cells stain positive for annexin V-PE and are negative for 7-AAD (Q4). Data are representative of three independent experiments.

Injection of a zPHF8 morpholino causes apoptosis in the zebrafish brain, which can be rescued by reintroduction of wild-type PHF8 [11]. Therefore, PHF8 may have a suppressive effect on the apoptosis of ESCC cells. We performed an annexin V-PE and 7-AAD assay to detect apoptosis. Knockdown of PHF8 significantly induced apoptosis of TE-1 and TE-2 cells, but not TE-8 cells (Figure 3). After PHF8 knockdown, the percentages of early- and late-stage apoptotic TE-1 cells were increased by 6- and 2-fold, respectively, whereas those of early- and late-stage apoptotic TE-2 cells were increased by 1.7- and 1.3-fold, respectively.

### PHF8 Promotes Migration and Invasion of ESCC Cells *in vitro*

Cancer cells acquire certain biological capabilities such as invasion and metastasis during the multistep development of tumors. ESCC is one of the most aggressive cancers, and its metastasis is a critical determinant of a poor prognosis. Thus, it is important to explore the effect of PHF8 on the migration and invasion of ESCC cells. Numerous *in vitro* systems have been developed to assess the invasiveness of tumor cells. In this study, we used two types of modified Boyden chamber to investigate the migration and invasion of the ESCC cell lines.

**Figure 4:**
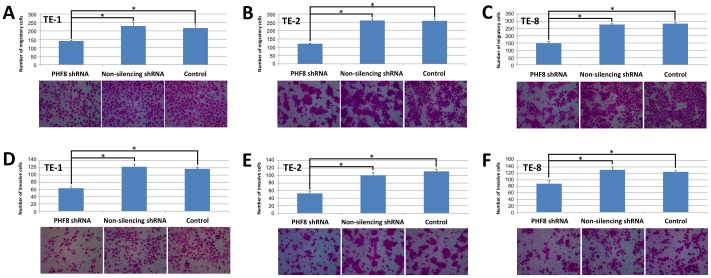
PHF8 enhances ESCC cell migration and invasion. ESCC cells stably expressing PHF8 or non-silencing shRNA, or control cells were seeded in upper chambers for migration and invasion assays. At the end of the experiments, migratory or invasive cells on the bottom surface were fixed, stained with H&E, and counted under a microscope at 200×. Data are the means ± SD of three independent experiments (*P<0.05). Representative images are shown. (**A**, **B** and **C**) Results of the migration assay of TE-1, TE-2, and TE-8 cells, respectively. (**D**, **E** and **F**) Results of the invasion assay of TE-1, TE-2, and TE-8 cells, respectively.

First, we used a chamber containing a polyethylene terephthalate membrane filter to examine the migration of ESCC cells. As expected, knockdown of PHF8 resulted in significantly fewer migratory cells in all of the studied ESCC cell lines (Figure 4A–C). The average numbers of migratory cells in the non-silencing shRNA group ranged from 229.44±19.08 to 277.77±7.29, which were comparable to those in the control group. However, the average numbers of migratory cells in the PHF8 shRNA group were reduced by nearly half (Figure 4A–C, Table S2A).

Compared with migration, invasion is more closely related to the process of metastasis because invasive cells need to breakdown the extracellular matrix before transversing membranes. Thus, we used a modified Boyden chamber containing a polyethylene terephthalate membrane coated with matrigel to investigate the invasion of ESCC cells. As a result, all of the tested ESCC cell lines were able to invade the coated membrane, but knockdown of PHF8 significantly reduced the invasive ability of all cell lines (Figure 4D–F). The average numbers of invasive cells in the three cell lines are shown in Table S2B.

### Downregulation of PHF8 Inhibits the Tumorigenicity of ESCC Cells *in vivo*

Our above *in vitro* studies indicate that PHF8 plays an important role in controlling ESCC cell proliferation, apoptosis, and colony formation *in vitro*. We therefore performed animal experiments using TE-1 cells to further investigate the role of PHF8 in the pathogenesis of ESCC *in vivo*.

**Figure 5:**
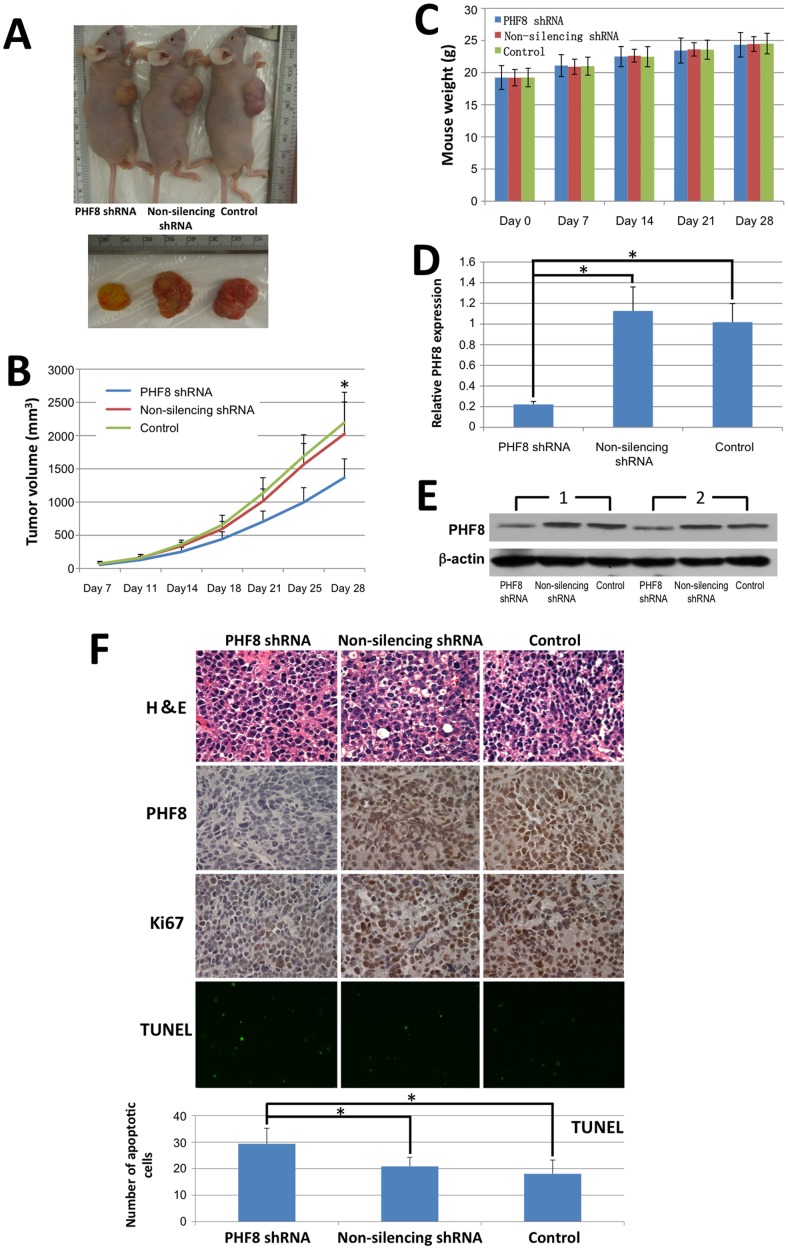
PHF8 supports ESCC cell growth *in vivo*. TE-1 cells stably expressing PHF8 or non-silencing shRNA, or control cells were injected subcutaneously into the right posterior flank of nude mice. Mice were sacrificed at 28 days after injection of the cells. (**A**) Representative images of subcutaneous tumors formed in nude mice. (**B**) Tumor growth curves. Data are the means ± SD (n = 7) (*P<0.05). (**C**) Histograms showing average mouse weights ± SD per group at the designated time points (n = 7). (**D** and **E**) Real-time quantitative PCR and western blot results showing the stability of PHF8 knockdown in tumors. Western blot analyses of two mouse tumor samples (1 and 2) from each group are shown. (**F**) H&E staining, immunohistochemical staining for PHF8 and Ki67, and TUNEL staining for apoptotic cells in tumors. Bar graph shows the number of TUNEL-positive cells (n = 7) (*P<0.05).

We found that tumors in nude mice formed by injection of TE-1 cells with knockdown of PHF8 expression were smaller compared with those formed by TE-1 cells expressing non-silencing shRNA or control cells (Figure 5A). The average volume of tumors in mice injected with cells expressing PHF8 shRNA was 1367.93±281.05 mm^3^ compared with 2026.00±479.75 mm^3^ and 2197.86±453.57 mm^3^ in mice injected with cells expressing non-silencing shRNA and control cells, respectively (*P = *0.047 and *P = *0.010, respectively) (Figure 5B). However, no significant weight differences were observed in mice among the three groups (Figure 5C). Knockdown persistence to the end of the experiment was verified by real-time quantitative PCR and western blot analyses of PHF8 expression in tumor tissues. In tumors derived from cells expressing non-silencing shRNA or control cells, PHF8 expression was high, whereas an obvious reduction of PHF8 expression was observed in tumors derived from PHF8 shRNA-expressing TE-1 cells (Figure 5D and 5E). The reduction of PHF8 expression in PHF8 shRNA-expressing TE-1 cells was further verified by immunohistochemistry using an anti-PHF8 antibody (Figure 5F).

Ki67 is a nuclear protein that is expressed in proliferating cells, and has been used as a marker for cell proliferation of solid tumors. In immunohistochemistry, we detected Ki67 expression in tumors of all three groups. Ki67 expression in tumors derived from PHF8 shRNA-expressing TE-1 cells was distinctly lower than that in tumors derived from cells expressing non-silencing shRNA or control cells (Figure 5F). Next, to evaluate apoptosis in ESCC tumors, we performed a terminal deoxynucleotidyl transferase-mediated deoxyuridine triphosphate nick-end labeling (TUNEL) assay to detect DNA fragmentation resulting from apoptotic signaling cascades (Figure 5F). The mean number of TUNEL-positive cells in tumors derived from PHF8 shRNA-expressing TE-1 cells was 29.37±5.90 compared with 20.86±3.40 and 18.06±5.21 in tumors derived from cells expressing non-silencing shRNA and control cells, respectively (*P* = 0.0222 and *P = *0.0078, respectively). Taken together, the above results suggest that PHF8 also plays a critical role in ESCC cell proliferation and apoptosis in tumors *in vivo*, which is in line with our *in vitro* results.

## Discussion

Previous studies on the carcinogenesis of ESCC have mainly focused on genetic alterations including those in COX-2, BCL-2, p53, p16, cyclin D1, EGFR, VEGF, and E-cadherin [4], [18]. It is well known that carcinogenesis is a multistage process in which genetic and epigenetic changes lead to oncogene activation and tumor suppressor gene inactivation [19]. Epigenetics is defined as heritable changes in gene expression, which are not accompanied by changes in DNA sequences, including DNA methylation, histone modifications, and non-coding RNAs [3]. Aberrant DNA methylation of tumor suppressor genes is well documented in ESCC. Our previous studies have shown that aberrant DNA methylation of the regulatory regions of human COX-2, SFRP1, SFRP2, and PTX3 genes plays a crucial role in the development of ESCC [4], [5], [20]. Non-coding RNAs are involved in a wide variety of biological and pathological processes including cell differentiation, proliferation, apoptosis, and metabolism. Of note, miR-21, miR-93, and miR-10a have been reported to be linked to ESCC pathogenesis [21]. Histone modifications comprise a multitude of covalent reactions affecting the histone N-terminal tails, including methylation, acetylation, phosphorylation, and ubiquitination at multiple and specific sites. Histone methylation has gained prominence because of its central roles in transcriptional regulation and other genomic functions [22], [23]. It is a reversible modification regulated by site-specific histone methyltransferases and KDMs. Overexpression/mutation of KDMs has been implicated in tumor initiation and progression [7]. In the present study, we investigated the role of PHF8, a member of the most recently discovered family of KDMs, in the pathogenesis and progression of ESCC.

Our results demonstrated many oncogenic features of PHF8 in ESCC, including inhibition of ESCC cell apoptosis as well as promotion of ESCC cell proliferation *in vitro* and tumor growth *in vivo*. We also found that PHF8 promoted ESCC cell migration and invasion, indicative of the potential involvement of PHF8 in promotion of tumor metastasis. In line with our results, there are reports showing that knockdown of PHF8 remarkably inhibits the proliferation of HeLa cancer cells [10], [11]. These studies have demonstrated that PHF8 is recruited to promoters and controls G1/S phase transition in conjunction with E2F1, HCF-1, and SET1A, partly through its KDM activity. Furthermore, it has been reported that PHF8 is key regulator of oncogenic functions of NOTCH1 signaling in T cell acute lymphoblastic leukemia (T-ALL), which is also dependent on its KDM activity [14]. Recently, PHF8 was found to have an effect on prostate cancer cell proliferation, migration, and invasion, in which the integrin pathway is linked to its oncogene function in human prostate cancer [16]. Moreover, this integrin signaling pathway was recently found to be involved in MLL-AF9 leukemia fusion-mediated leukemic transformation of bone marrow cells [24]. Therefore, these elegant studies raise the possibility that the integrin signaling pathway via a PHF8-dependent manner may also play important roles in the pathogenesis of ESCC, which needs to be studied further.

In this study, we observed different oncogenic effects in the three ESCC cell lines following downregulation of PHF8 expression. Inhibitive effects on cell proliferation, anchorage-dependent growth, migration, and invasion were observed in all three ESCC cell lines with PHF8 knockdown. However, the inhibitive effects on anchorage-independent growth and induction of apoptosis were not observed in TE-8 cells with PHF8 knockdown. The ESCC cell lines used in our study have different genetic backgrounds and tumor stages. TE-1 cells are well differentiated and the TNM Classification of Malignant Tumors (TNM) stage is T3N0M0 stage 2a, whereas TE-2 cells are poorly differentiated with a TNM stage of T4N1M0 stage 3, and TE-8 cells are moderately differentiated with a TNM stage of T3N1M0 stage 3. Therefore, the distinct PHF8 oncogenic effects in these ESCC cell lines may be linked to their genetic and epigenetic backgrounds.

Recent studies have shown that overexpression of LSD1 histone demethylase is linked to the pathogenesis of many types of cancers including breast, prostate, and lung cancers [25], [26]. Moreover, the development of small molecule inhibitors of LSD1 has resulted in their application to inhibit tumor cell growth [27]. Overexpression or mutation of JmjC domain-containing KDMs has also been linked to many types of cancer, including PHF8 in prostate cancer [16]. Therefore, small molecules targeting these KDMs may be of great interest as novel anti-tumor agents [8]. However, the current KDM inhibitors have been designed to inhibit binding of the alpha-ketoglutarate co-factor to the JmjC enzymatic domain of KDMs. Therefore, these small molecules may also inhibit other important enzymes that require alpha-ketoglutarate as a co-factor for their enzymatic activities, such as TET2 (ten-eleven translocation 2) hydroxylase, which has a tumor suppressor function [28]. Thus, these inhibitors need to be further refined to increase their specificity for greater utility.

We previously showed that PHF8 interacts with RARα and functions as a coactivator for RARα in a KDM-activity dependent manner and plays a critical role in neuronal differentiation [12]. Very recently, we demonstrated that the enzymatic activity of PHF8 is linked to the retinoic acid sensitivity in acute promyelocytic leukemia (APL) [29]. The present study suggests the potential molecular manipulation of PHF8 activity in a phosphorylation-dependent manner in ESCC, which could be an alternative strategy for regulation of PHF8 activity and serve as a potential and efficient treatment of ESCC.

Taken together, our data support the important role of PHF8 in ESCC and suggest that PHF8 may be a target for treatment/prevention of ESCC. Future studies should focus on the molecular mechanisms of PHF8-induced ESCC tumor development and progression as well as on strategies to downregulate this important epigenetic protein or inhibit its function for a potential therapeutic strategy in ESCC [8].

## Materials and Methods

### Cell Culture

ESCC cell lines TE-1, TE-2, and TE-8, which have been described previously [4], [30], were cultured in RPMI 1640 medium (Life Technologies, Carlsbad, CA) supplemented with 10% fetal bovine serum (FBS; Life Technologies), 100 U/ml penicillin, and 100 µg/ml streptomycin (Life Technologies). HEK293T cells were cultured in Dulbecco’s modified Eagle’s medium (Life Technologies) supplemented with 10% FBS, 100 U/ml penicillin, and 100 µg/ml streptomycin. All cells were cultured at 37°C in a humidified atmosphere with 5% CO_2_.

### Lentivirus Preparation and Infection

pGIPZ lentiviral vectors carrying a GFP reporter gene and shRNA sequence specific for PHF8 or non-silencing shRNA were purchased from Thermo (Rockford, IL). Two different shRNAs targeting PHF8 were used: shPHF8 (ACTATGTTGGTTCTGACAA) and shPHF8-2 (CGGCGAACCAAGATAGCAA). HEK293T cells were transfected with packaging, envelope, and shRNA transfer vectors. After 48–72 h of transfection, the medium containing the lentivirus was collected and concentrated by ultracentrifugation. HEK293T cells were infected with the diluted lentivirus, and then GFP expression was confirmed under a fluorescence microscope at 48 h post-infection. We then counted GFP-expressing colonies as one transduced cell and the viral titer (transducing units per milliliter) was calculated using the formula: number of GFP-positive colonies×dilution factor×40. The diluted virus was then used to infect ESCC cell lines to establish cells constitutively repressing PHF8 expression. Briefly, 40–60% of confluent ESCC cell cultures were infected at an appropriate multiplicity of infection, incubated for 6 h, and then the medium was replaced with fresh medium. At 48 h post-infection, stable clones were selected with 1 µg/ml puromycin (Sigma-Aldrich, St. Louis, MO).

### Real-time Quantitative RT-PCR

Total RNA was extracted from cells or tumor tissues using an RNeasy Mini Kit (Qiagen, Valencia, CA) according to the manufacturer’s instructions [17], [31], and then treated with DNase I (Qiagen). cDNA was synthesized from 500 ng of total RNA using iScript^TM.^ Reverse Transcription Supermix (Bio-Rad, Hercules, CA). Quantitative real-time PCR was performed using 40 ng of cDNA template, RT^2^ SYBR® Green qPCR Mastermix (Qiagen), and 30 nM each of the appropriate primers (Life Technologies). The GAPDH gene was used as an endogenous control. Primers used in this study were as follows: PHF8, 5′-GACATGTGCCAGGACTGGTTT-3′ and 5′-CAGCAGCCTTCTCCTCTTCAA-3′; GAPDH, 5′-CCACATCGCTCAGACACCAT-3′ and 5′-GCGCCCAATACGACCAAAT-3′. PCR was carried out using an ABI 7300 Real-Time PCR System (Applied Biosystems, Carlsbad, CA), and the cycling conditions were denaturation at 95°C for 10 min, followed by 40 cycles of 95°C for 15 s and 60°C for 1 min. Each sample was run in triplicate for three independent experiments. Relative mRNA levels were determined by the 2^−ΔΔCt^ method as described previously [31].

### Western Blot Analysis

Cells or animal tissues were lysed in RIPA buffer (50 mM Tris-HCl pH 8.0, 150 mM NaCl, 1% NP-40, 0.5% sodium deoxycholate, and 0.1% SDS) containing Protease Inhibitor Cocktail Tablets (Roche, Indianapolis, IN) as described previously [12]. Equal amounts of total protein were fractionated by 7.5% SDS-polyacrylamide electrophoresis and then transferred to polyvinylidene fluoride membranes (Millipore, Billerica, MA). The membranes were incubated with an anti-PHF8 antibody (1∶2500; Abcam, Cambridge, MA) at 4°C overnight, followed by a horseradish peroxidase-conjugated secondary antibody (1∶5000) for 1 h at room temperature. β-actin served as the loading control and was detected using a mouse anti-β-actin monoclonal antibody (Sigma-Aldrich).

### Cell Proliferation Analysis

Cells were plated onto a 96-well plate at a density of 1×10^3^ cells per well. Viability of the cells was measured using a CellTiter 96® Aqueous One Solution Cell Proliferation Assay (Promega, Madison, WI) as described previously with modifications [17], [20]. After 0, 24, 48, 72, and 96 hours, the optical density at 492 nm was measured using a Multiskan Ascent® microplate photometer (Thermo). Three independent experiments were performed in triplicate.

### Cell Apoptosis Analysis

Apoptosis was measured using an Annexin V-PE/7-AAD Kit (Becton Dickinson, Franklin Lakes, NJ) following the manufacturer’s instructions [17]. Briefly, cells were collected, washed with cold PBS, and resuspended in Binding Buffer at a final concentration of 1×10^6^ cells/ml. Cells (1×10^5^) were incubated with 5 µl of annexin V-PE and 5 µl of 7-AAD for 15 minutes at 25°C in the dark. Then, 400 µl of Binding Buffer was added to each tube, and the cells were analyzed by a BD LSRFortessa cell analyzer (Becton Dickinson) within 1 h. A minimum of three independent experiments was performed.

### Colony Formation Assay

To evaluate anchorage-dependent colony formation, 500 cells were suspended in 3 ml of RPMI 1640 medium, plated in a well of six-well plate, and then incubated at 37°C in a humidified atmosphere with 5% CO_2_ for 10–14 days or when colonies appeared. Then, the cells were fixed with methanol and stained with 1 ml of Giemsa (Sigma-Aldrich) to count the colonies. The experiment was performed in at least triplicate wells and repeated three times.

### Soft Agar Assay

Anchorage-independent colony formation was examined using a soft agar assay. Two thousand cells were suspended in 1 ml of 0.35% low-temperature melting agarose dissolved in RPMI 1640 medium, plated on top of 0.7% solidified agarose in a well of a six-well plate, and then incubated at 37°C in a humidified atmosphere with 5% CO_2_ for 10–14 days or when colonies appeared. Then, the cells were stained with 0.3 ml of 0.05% p-iodonitrotetrazolium violet (Sigma-Aldrich_)_ at 37°C overnight, and the visible colonies were counted. The experiment was performed in at least triplicate wells and repeated three times.

### *In vitro* Migration and Invasion Assays

*In vitro* migration assays were performed using a BD chamber (Becton Dickinson) containing a polyethylene terephthalate membrane filter (6.4-mm diameter; 8-um pore size). *In vitro* invasion assays were performed using a BD BioCoat^TM.^ Matrigel^TM.^ Invasion Chamber (Becton Dickinson) containing a polyethylene terephthalate membrane filter (6.4-mm diameter; 8-um pore size) precoated with a thin layer of matrigel. Briefly, the upper chamber was seeded with the appropriate number of cells (0.5×10^5^ of TE-1 cells; 1×10^5^ of TE-2 or TE-8 cells) suspended in 0.5 ml of serum-free RPMI 1640 medium. The lower chamber contained 0.75 ml of complete RPMI 1640 medium. After 24–36 h of incubation at 37°C in a humidified atmosphere with 5% CO_2_, non-migratory or non-invasive cells on the upper surface of the membrane were wiped off. Migratory or invasive cells on the bottom surface were fixed with methanol, stained with hematoxylin and eosin (H&E), and counted under a microscope.

### Xenograft Studies of Nude Mice

TE-1 cells were selected to perform the nude mouse xenograft study. Four-week-old female athymic nude mice were obtained from Charles River Laboratories (Wilmington, MA) and maintained under specific pathogen-free conditions according to the institutional guidelines for the care and use of laboratory animals. All animal studies were approved by the Institutional Animal Care and Use Committee of Baylor College of Medicine. All surgical procedures were performed under isoflurane anesthesia, and all efforts were made to minimize suffering. Mice were randomized into three groups with seven animals in each group: PHF8 shRNA, non-silencing shRNA, and untreated control groups. Cells (4×10^6^) were suspended in 100 µl of serum-free RPMI 1640 medium. The mice were anesthetized with isoflurane and then TE-1 cells were injected subcutaneously into their right posterior flank. Tumor diameters were measured at regular intervals with digital calipers, and the tumor volume in cubic millimeters was calculated using the formula: volume = (width)^2^×length/2. A tumor growth curve was constructed, and data were presented as means ± SD. Animals were sacrificed be cervical dislocation after placing the mice under isoflurane anesthesia at 28 days after injection of the cells. Tumors were dissected, weighted, and frozen in liquid nitrogen or fixed in 10% neutral buffered formalin for further analysis.

### Immunohistochemistry

Fixed tissues were embedded in paraffin for sectioning. Five micrometer-thick paraffin sections were deparaffinized and heated for antigen retrieval in sodium citrate buffer. Then, endogenous peroxidases were inactivated with 3% hydrogen peroxide and non-specific binding sites were blocked with 10% normal goat serum for 1 h. The sections were then incubated with the following primary antibodies (both purchased from Abcam): rabbit polyclonal anti-PHF8 (1∶200) or anti-Ki67 (1∶200) at 4°C overnight, followed by a biotinylated secondary antibody for 30 min. An avidin-biotin-peroxidase complex was added to the sections and incubated for 1 h. Color was developed using DAB substrate. The sections were then counterstained with hematoxylin, dehydrated, and mounted with DPX.

### TUNEL Assay

Apoptotic cells in tumor tissues from nude mice were detected by the TUNEL method using a commercially available TUNEL kit (Millipore, Billerica, MA) according to the manufacturer’s instructions. 4,6-Diamidino-2-phenylindole was used for nuclear counterstaining. One representative section was selected for each mouse, and TUNEL-positive cells in five random fields were counted and averaged for each section.

### Statistical Analysis

All statistical analyses were performed using SPSS software. The results are presented as the means ± SD. Differences between groups were assessed by analysis of variance or a t-test. A value of *P*<0.05 was considered statistically significant.

## Supporting Information

Figure S1 **PHF8 promotes ESCC cell proliferation.** ESCC cell lines TE-1 (**A**), TE-2 (**B**), and TE-8 (**C**) stably expressing PHF8 shRNA-2 or non-silencing shRNA, or control cells were cultured with MTS and analyzed using a microplate photometer at the indicated times. Standard deviation bars were obtained from three independent experiments (*P<0.05). Downregulation of PHF8 mRNA and protein expression induced by PHF8 shRNA-2 was confirmed by real-time quantitative PCR and western blotting, respectively, in which, GAPDH and β-actin served as endogenous controls, respectively.
(TIF)

Table S1 **Related to** **Figure 2** **.** (**A**) Number of colonies in plate colony formation assay. (**B**) Number of colonies in soft agar colony formation assay.
(TIF)

Table S2 **Related to** **Figure 4** **.** (**A**) Number of migratory cells in migration assay. (**B**) Number of invasive cells in invasion assay.
(TIF)
