# Supplementary figures and images for: Oncogenic Features of PHF8 Histone Demethylase in Esophageal Squamous Cell Carcinoma
Source: PLoS One. 2013 Oct 11;8(10):e77353. doi: 10.1371/journal.pone.0077353 (PMC3795633; doi:10.1371/journal.pone.0077353)

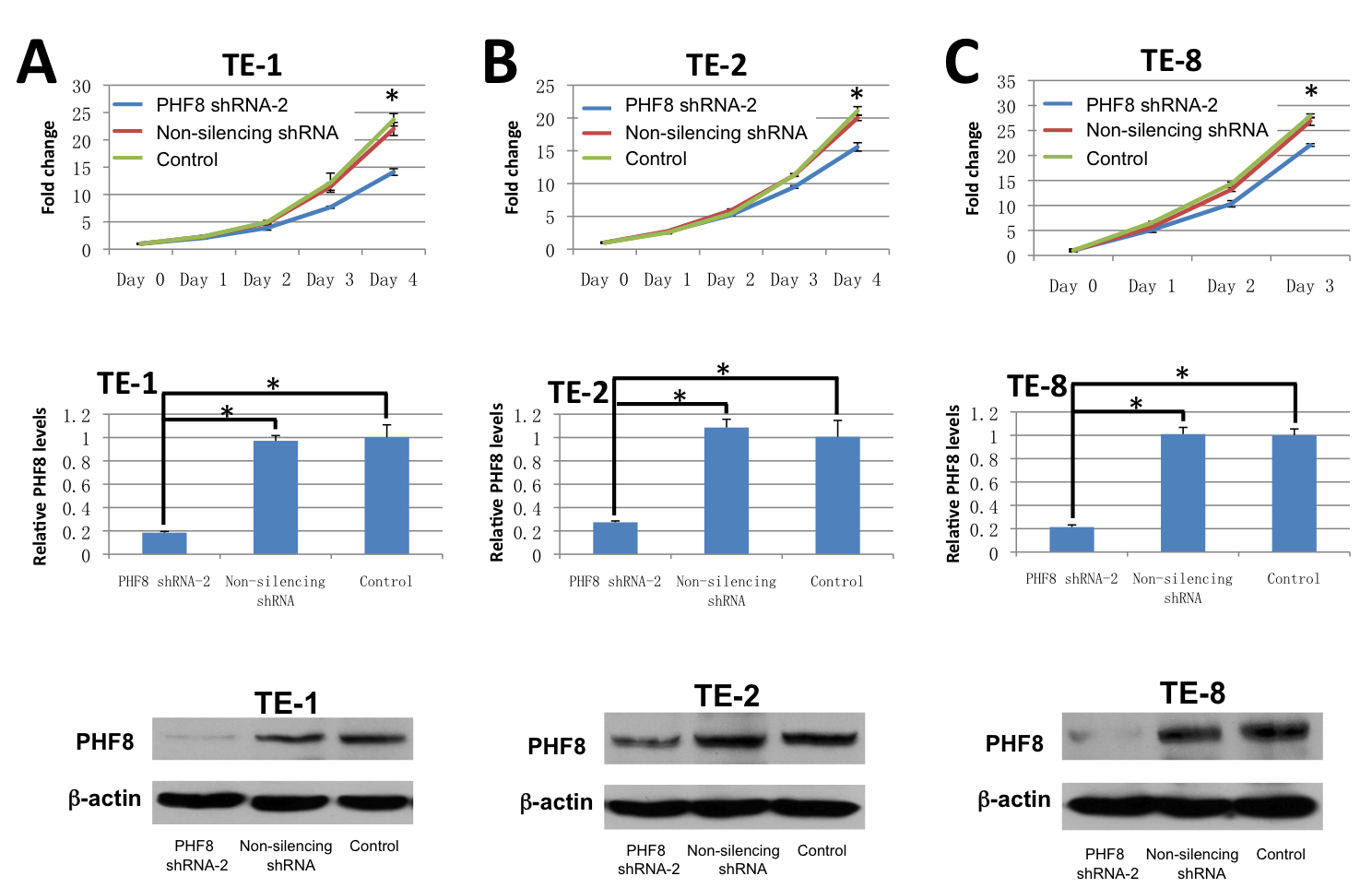

Supplement: Figure S1 — PHF8 promotes ESCC cell proliferation. ESCC cell lines TE-1 (A), TE-2 (B), and TE-8 (C) stably expressing PHF8 shRNA-2 or non-silencing shRNA, or control cells were cultured with MTS and analyzed using a microplate photometer at the indicated times. Standard deviation bars were obtained from three independent experiments (*P<0.05). Downregulation of PHF8 mRNA and protein expression induced by PHF8 shRNA-2 was confirmed by real-time quantitative PCR and western blotting, respectively, in which, GAPDH and β-actin served as endogenous controls, respectively. (TIF) [file pone.0077353.s001.tif]

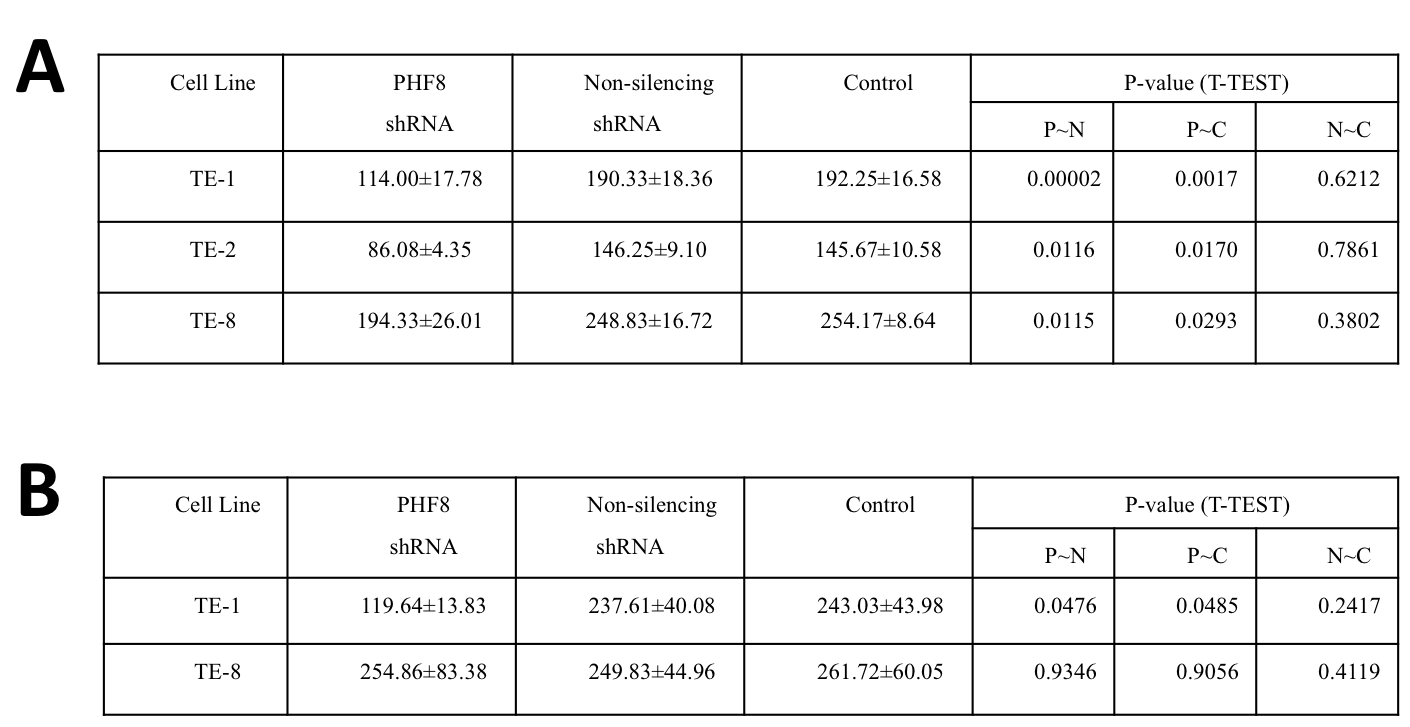

Supplement: Table S1 — Related to Figure 2 . (A) Number of colonies in plate colony formation assay. (B) Number of colonies in soft agar colony formation assay. (TIF) [file pone.0077353.s002.tif]

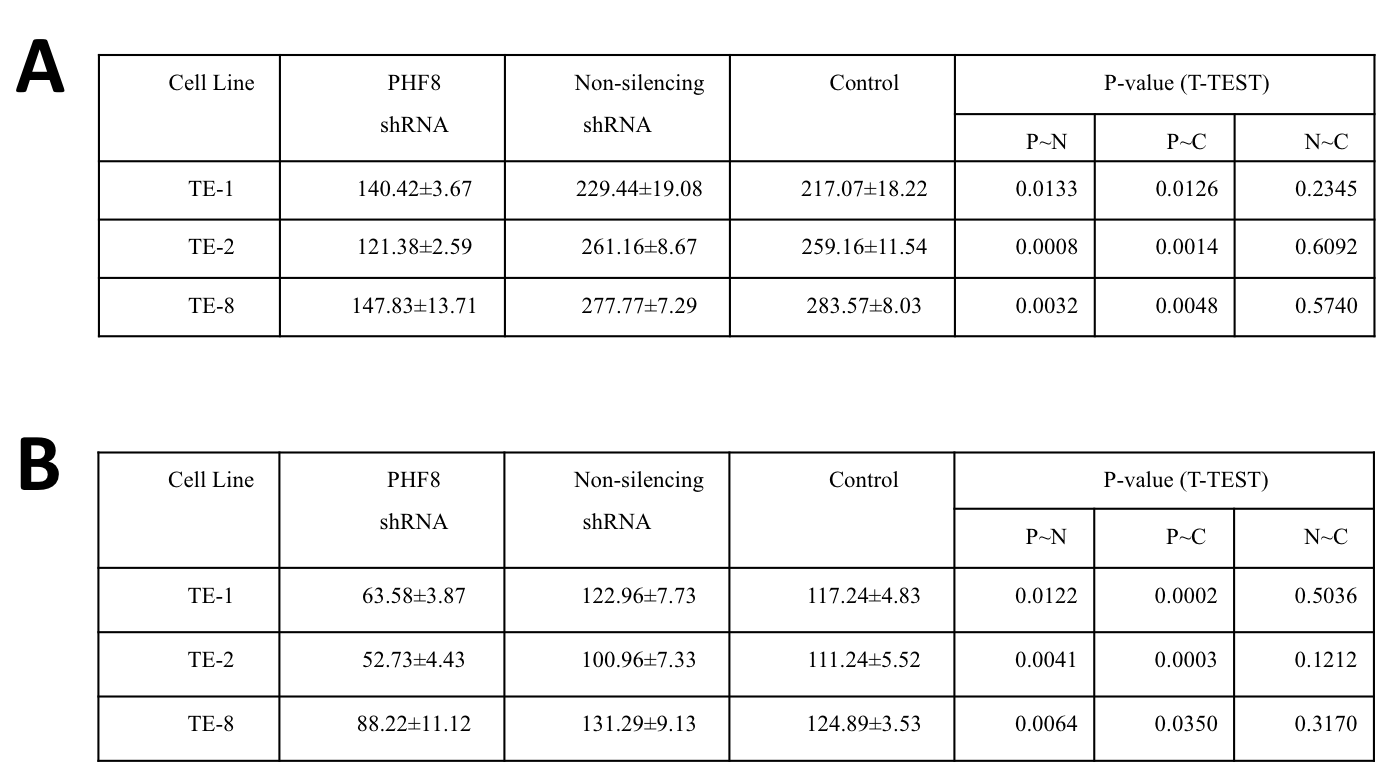

Supplement: Table S2 — Related to Figure 4 . (A) Number of migratory cells in migration assay. (B) Number of invasive cells in invasion assay. (TIF) [file pone.0077353.s003.tif]
